# Structure and Mechanical Properties of Ti-38Zr-(8-10)Nb (at. %) Alloys for Medical Use

**DOI:** 10.3390/jfb17040179

**Published:** 2026-04-03

**Authors:** Konstantin V. Sergienko, Sergei V. Konushkin, Yaroslava A. Morozova, Maria A. Sudarchikova, Mikhail A. Kaplan, Vadim K. Zhidkov, Tatyana M. Sevostyanova, Aleksander V. Simakin, Ilya V. Baimler, Mikhail A. Sevostyanov, Alexey G. Kolmakov

**Affiliations:** 1 A.A. Baikov Institute of Metallurgy and Materials Science, Russian Academy of Sciences (IMET RAS), 119334 Moscow, Russia; venev.55@mail.ru (S.V.K.); yasya12987@gmail.com (Y.A.M.); mariahsudar@yandex.ru (M.A.S.); mishakaplan@yandex.ru (M.A.K.); vkzhidkov@yandex.ru (V.K.Z.); cmakp@mail.ru (M.A.S.); akolmakov@imet.ac.ru (A.G.K.); 2 National Medical and Surgical Center Named after N.I. Pirogov of the Ministry of Health of the Russian Federation, 117513 Moscow, Russia; tata_sev1048@mail.ru; 3 Moscow Regional Research and Clinical Institute (MONIKI), 129110 Moscow, Russia; 4 Prokhorov General Physics Institute of the Russian Academy of Sciences, Vavilov Street 38, 119991 Moscow, Russia; avsimakin@gmail.com (A.V.S.);

**Keywords:** Ti-Zr-Nb, β-Ti, superelasticity, Young’s modulus, mechanical properties, biocompatible materials

## Abstract

The research described in this article is a continuation of a series of studies on biocompatible materials, focused on finding the optimal alloy composition and heat treatment regimes. The use of materials with a low Young’s modulus ensures the long-term safety of the implant by reducing the stress shielding effect, which causes bone resorption. This work investigates the effect of alloying with niobium in the range of (8–10) at. % on the Ti-38Zr alloy, specifically its structure, mechanical properties, Young’s modulus, and superelasticity. In this study, plates of the Ti-38Zr-(8-10)Nb (at. %) alloy were investigated after quenching and subsequent annealing. In Ti-38Zr-(8-10)Nb alloys, quenching from 600 °C fixes the β-phase of Ti. In alloys with (8-9)Nb, this is a metastable β-phase, as evidenced by its superelastic behavior under cyclic tension. Annealing at 400 °C leads to a clear decomposition of the quenched high-temperature β-phase in Ti-38Zr-(8-9)Nb alloys into β- and α′-phases. Based on the mechanical test results, it can be inferred that the precipitation of the brittle ω-phase and the α′-phase occur concurrently, since annealing at 400 °C causes a pronounced embrittlement of the Ti-38Zr-(8–9)Nb alloys (with elongation dropping from ~15% to 0.7–2.5%, respectively) alongside a substantial increase in strength (from 500 MPa to 1010 MPa). For the Ti-38Zr-10Nb alloy, the ductility also declines but remains within acceptable limits (from ~14% to ~10%), while the strength rises from 520 MPa to 630 MPa. The Young’s modulus of the Ti-38Zr-(8-10)Nb alloy after quenching is ~80 GPa. After annealing, it increases to 95 GPa for alloys with (8-9)Nb, while for 10Nb it remains at approximately 80 GPa.

## 1. Introduction

Titanium alloys are now extensively employed in implant fabrication owing to their superior biocompatibility, favorable mechanical characteristics, resistance to corrosion, and elastic moduli that are comparatively lower than those of stainless steels and Co-Cr-Mo alloys [1].

Commercially pure titanium and the Ti-6Al-4V alloy (Grade 5) rank among the most frequently used titanium alloys in medical applications. A mismatch in Young’s modulus between the implant and adjacent bone tissue can, however, induce stress shielding at the implantation site, which in turn leads to bone weakening and atrophy, ultimately necessitating revision surgery [2,3].

Alloys with a superelasticity effect, particularly nitinol (titanium nickelide), are widely used in bone plates and orthodontic structures due to their ability to withstand significant cyclic loads. The potential toxicity of nickel (a constituent of nitinol) and of aluminum and vanadium ions (from Ti-6Al-4V) poses a challenge for these materials [4,5,6,7].

The development of advanced titanium alloys for medical purposes requires ensuring high mechanical properties, a low elastic modulus, and a safe chemical composition. Alloys based on the metastable BCC phase (β-Ti), which have lower Young’s moduli compared to α- and (α + β)-titanium alloys, are of great interest to researchers in the field of biomedical implants. To date, the most promising biocompatible β-stabilizing elements are zirconium, niobium, tantalum, and molybdenum; consequently, alloys based on these elements are being actively developed [2,3,8].

Zirconium acts as a neutral element with respect to titanium and its alloys. It has a slight stabilizing effect on the β-phase of titanium at contents below 10 at. %. However, above this threshold, zirconium begins to more actively stabilize β-titanium [2,9]. Numerous studies have been conducted on the development of biomedical alloys based on the Ti-Zr system [10,11,12,13,14].

Ti-Zr alloys additionally alloyed with Nb have enormous potential as implantable medical devices (e.g., endoprostheses) and are currently being extensively studied [15,16,17,18]. In some elemental combinations, these alloys exhibit superelasticity, which is attributed to the martensitic transformation β↔α’’ upon loading and unloading. Previous studies have shown the most balanced zirconium content to be 38 at. % with a Nb content of 9 at. %. The examined alloy exhibited a uniform microstructure composed of polyhedral β-Ti grains, stabilized by niobium and zirconium. Quenching proved advantageous, leaving the Young’s modulus, microhardness, and strength properties virtually unchanged while improving ductility and promoting a more pronounced superelastic response. The resulting elastic modulus of 50–60 GPa closely approaches that of human cortical bone. In comparison to alloys containing 36 and 40 at. % Zr, this composition demonstrated superior static mechanical characteristics, including an ultimate tensile strength of approximately 630–660 MPa and an elongation of 14–23%, along with enhanced superelastic behavior [16]. Although the Ti–38Zr–11Nb alloy investigated in [17] exhibited a lower Young’s modulus than pure titanium and Grade 5, its modulus still exceeded 80 GPa when measured by nanoindentation. This is associated with the formation of a stable β-Ti phase, whereas the desired microstructure consists of the metastable β-Ti phase, which provides a lower Young’s modulus.

Data from Refs. [19,20] were used to select the zirconium content. To determine the appropriate niobium content for the chosen zirconium concentration, the molybdenum equivalent [Mo]eq [21], the mean valence electron concentration per atom (e/a) [22,23], and the approach using the Bo and Md parameters [24] were employed. The molybdenum equivalent is commonly used as a guideline in the development of titanium alloys: if the [Mo]eq is approximately 10% or higher, the alloy will retain the β-phase after water quenching. The following formula was used to calculate [Mo]eq [21]:

[Mo]eq= 0.97 + 0.238·Nb(mass %)+ 0.11·Zr(mass %)

As an alternative indicator of β-phase stability, the average number of valence electrons per atom (e/a ratio) was used. This ratio is widely used to predict the elastic properties of β-Ti alloys, as a well-established correlation shows that Young’s modulus decreases with a lower e/a ratio [23].

e/A = 4·Ti(at.%) + 4·Zr(at.%) + 5·Nb(at.%)

The evaluation of the alloy also involved the Bo and Md parameters, where Bo denotes the bond order and Md the metal d-orbital energy level. Calculations were performed using the expressions provided in [24]:

$$\bar{Md}=\sum_{i}X_{i}\;*\;{Md}_{i}$$

$$\bar{Bo}=\sum_{i}X_{i}\;*\;{Bo}_{i}$$

where X_i_ is the atomic fraction of the i-th element, and Bo_i_ and Md_i_ are tabulated values for each element.

These two parameters are used as the axes on the Bo–Md diagram: the position of the alloy on this diagram makes it possible to predict whether the β-phase will be stable, or whether ω, α″ phases, etc., will appear [24].

Based on the described methods, low-modulus alloys with compositions of Ti-38Zr-(8-10)Nb (at. %) were selected and obtained. For example, for the Ti-38Zr-9Nb alloy, the calculation of the molybdenum equivalent [Mo]_eq_, the e/a ratio, and the Bo and Md values is as follows:

$${\left[Mo\right]}_{eq}=0.97\;+\;0.238\cdot 12.2\left(mass\%Nb\right)+\;0.11\cdot 50.68\left(mass\%Zr\right)=9.45$$

$$\frac{e}{A}=4\cdot 0.53\left(at\%Ti\right)+4\cdot 0.38\left(at.\%Zr\right)+5\cdot 0.09\left(at.\%Nb\right)=4.09$$

$$\bar{Md}=0.53\cdot 2.447+0.38\cdot 2.934+0.09\cdot 2.424=2.62999\approx 2.63$$

$$\bar{Bo}=0.53\cdot 2.790+0.38\cdot 3.086+0.09\cdot 3.099=2.93029\approx 2.93$$

Thus, according to the calculated values of the molybdenum equivalent [Mo]_eq_ [22], the e/a ratio [23], and the Bo/Md parameters [24], the chemical composition of the selected alloys should allow the β-phase to be retained in the structure after quenching.

## 2. Materials and Methods

### 2.1. The Object of Research

This study investigated plates of the Ti–38Zr–(8–10)Nb alloy (at.%), for which the titanium was supplied by PJSC VSMPO-AVISMA (Moscow, Russia) and the alloying elements by Chepetsky Mechanical Plant Stock Company (Glazov, Russia).

### 2.2. Production and Preparation of the Material

The melting and homogenization annealing process was carried out according to an established procedure for this class of materials [17]. Melting was carried out in a vacuum arc furnace equipped with a non-consumable electrode (L200DI, LeyboldHeraeus, Hanau, Germany). After evacuating the chamber to 1.33 Pa, argon was introduced until a pressure of 40 × 10^3^ Pa was reached. The metals were arranged in the crucible according to their melting points, and melting was initiated by an electric arc traveling from the top downward. Prior to melting the actual ingots, a getter placed in a separate well was melted first to further purify the argon atmosphere. Each ingot was re-melted seven times, with the sample being turned over between melts to ensure thorough mixing of the components. These repeated remelting steps were performed carefully to guarantee compositional uniformity throughout the entire volume of the ingot. The melting time per ingot was 1 to 1.5 min. The resulting ingot had a “boat” shape, measuring approximately 120 mm in length, 25 mm in width, and 12 mm in height.

Owing to the different melting points of the starting materials, the as-cast ingot exhibited a dendritic structure. To homogenize the composition, the alloy was annealed in a vacuum furnace (ESKVE-1.7.2.5/21 ShM13, NITTIN, Moscow, Russia) at 1000 °C for 4 h. A vacuum of 27 × 10^−4^ Pa was maintained during the heat treatment to prevent oxidation [17].

Hot rolling at 600 °C was used to produce plates of the selected alloys.

Rolling was performed in several passes to achieve the required thickness and shape.

Prior to deformation, the workpieces were heated in a KYLS 20.18.40/10 muffle furnace (HANS BEIMLER, Berlin, Germany) for 20–25 min before the first rolling pass and for 5 min during intermediate passes. The ingots, with an initial thickness of ~12 mm, were rolled on a DUO-300 two-high rolling mill (IMET RAS, Moscow, Russia) using the following reductions per pass: 1.5 mm per pass until the thickness reached 4.0 mm, then 1.0 mm per pass until the thickness reached 2.0 mm. Final rolling from 2 mm to 1 mm was carried out without preheating, with a reduction of 0.5 mm per pass. This was because the rolling mill was changed to a QUARTO model (IMET RAS, Moscow, Russia), and the equipment was not recommended for use with heated workpieces. Additionally, the absence of preheating reduces oxide layer formation on the plate surface. The energy accumulated during deformation (in the form of increased dislocation density and crystal lattice distortions) acts as an additional driving force, accelerating recrystallization and β-grain growth during subsequent heating for quenching. This allows for targeted control over the final grain size and homogeneity.

After rolling, the plates were quenched in water from the β-phase region, specifically from 600 °C. The samples were placed in a preheated furnace, held for 5 min, and then cooled in water at 20 °C.

Annealing was carried out at 400 °C for 1 h in a vacuum of 2.67 × 10^−3^ Pa, with heating and cooling rates of 10 °C/min.

Samples for testing were cut from the plates using electrical discharge machining (EDM) on a Meatek DK7745 unit (NPP “MEATEK”, Moscow, Russia).

Sample preparation for microstructural examination, X-ray diffraction (XRD), and nanoindentation followed a multi-step grinding and polishing procedure. The samples were sequentially ground using a Piatto diamond disc with grit sizes of P220 (10 min), P600 (10 min), P1000 (5 min), P1200 (5 min), P2500 (5 min), and P4000 (5 min). This was followed by polishing on an Akasel NAPAL velvet cloth using DiaMaxx Poly diamond suspensions with particle sizes of 1 μm and 50 nm, each applied for 5 min. A Phoenix 4000 polishing machine (Buehler, Lake Bluff, IL, USA) was employed throughout. The final surface exhibited a mirror-like finish.

### 2.3. Methods of Research

Nitrogen and oxygen concentrations were determined by inert gas fusion in a graphite crucible using a LECO TC-600 analyzer (LECO Corporation, 3000 Lakeview Avenue, St. Joseph, MI, USA), equipped with a pulsed resistance furnace, with helium as a carrier gas. Nitrogen quantification was performed using the thermal conductivity method, while oxygen was quantified as carbon dioxide (CO_2_) by infrared absorption spectroscopy.

Hydrogen content was determined by inert gas fusion in a graphite crucible using a LECO RHEN-602 analyzer (LECO Corporation, 3000 Lakeview Avenue, St. Joseph, MI, USA), equipped with a pulsed resistance furnace, with argon as a carrier gas. Hydrogen quantification was performed using the thermal conductivity method.

Sulfur and carbon contents were measured using an induction furnace with a flux, where combustion took place in a ceramic crucible, and analysis was performed with a LECO CS-600 analyzer (LECO Corporation, 3000 Lakeview Avenue, St. Joseph, MI, USA). Sulfur and carbon were detected as CO_2_ and SO_2_, respectively, by infrared absorption spectroscopy.

Elemental distribution was examined using a KYKY EM6900 scanning electron microscope (SEM) (KYKY TECHNOLOGY CO, Beijing, China) equipped with an Oxford Instruments EDS detector (Oxford Instruments, Abingdon, Oxfordshire, UK) and the accompanying AZtec software (v 6.0 SP2, Oxford Instruments, 2021).

X-ray diffraction (XRD) was conducted using a Haoyuan DX2700mini diffractometer (Dandong Haoyuan Instrument Co., Dandong, China). CuKα radiation (λ = 1.54178 Å) was employed, with data collected over a 2θ range of 20–100° at a step size of 0.01° and a counting time of 0.5 s per step. The X-ray source operated at 40 kV and 13 mA. Diffraction patterns were processed with HighScore Plus software (version 3.0.5, PANalytical, Almelo, The Netherlands, 2012).

For microstructural observation, sample surfaces were etched using a solution containing nitric acid, hydrofluoric acid, and distilled water in a volumetric ratio of HF: HNO_3_: 15H_2_O. The polished samples were immersed in the etchant for 5 to 30 s, followed by rinsing under running water.

Optical microscopy was performed on an Altami MET 5C microscope (Altami LLC, Saint Peterburg, Russia), which was fitted with a 14 MP high-resolution camera and operated using Altami Studio 4.0 software.

Mechanical testing was carried out on an INSTRON 3382 universal testing machine (Instron, Norwood, MA, USA). Tensile tests were run at a crosshead speed of 1 mm/min. The specimens, cut from the plates by electrical discharge machining, had a gauge length of 10 mm, a thickness of 1 mm, and a width of 2 mm. Test data were evaluated in accordance with Russian standard GOST 1497-84 using INSTRON Bluehill 2.0 software. For each treatment condition, at least nine specimens were tested.

Young’s modulus was measured with a NanoScan-4D nanohardness tester (NauchSpecPribor, Troitsk, Russia). Instrumented indentation was performed following ISO 14577-1:2002. A Berkovich diamond tip was used to apply a load of 500 mN to the matrix. Indentations were made across the full width of each specimen, with a loading/unloading rate of 20 mN/s, a hold time of 20 s, and a spacing of 200 μm between indentations. A total of 27 indentations were made per treatment condition. The resulting indentation curves were analyzed using NANOINDENTATION 3.0 software (CSM Instruments, Peseux, Switzerland), assuming a Poisson’s ratio of 0.3, and the results were averaged over five experimental curves. Elastic recovery was calculated as the ratio of elastic work to total indentation work. The maximum penetration depth of the indenter was approximately 3500 nm.

Fracture surfaces were examined using a KYKY EM6900 scanning electron microscope (SEM) equipped with a secondary electron (SE) detector at an accelerating voltage of 20 kV.

## 3. Results

### 3.1. Chemical Composition and Impurities

The compositions of the alloys according to the results of the EDS attachment study are presented in Table 1.

The plates were also tested for impurity content, with the results shown in Table 2.

Chemical analysis confirmed that the impurity content of the obtained alloys meets the specifications of GOST 19807-91 [25] for titanium alloys of the VT1-0 type.

### 3.2. Microstructure

**Figure 1:**
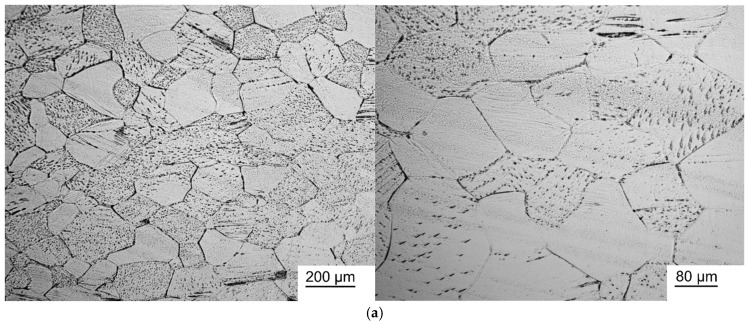

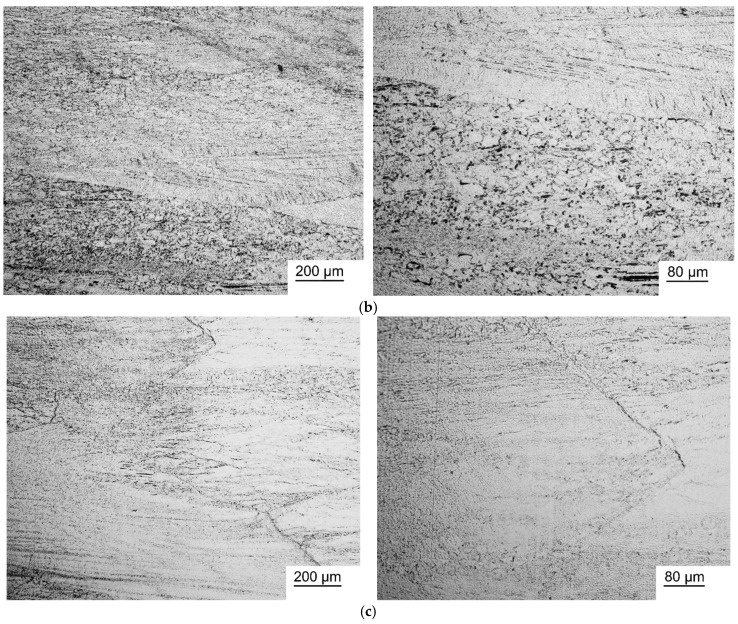
Microstructure of Ti-38Zr-(8-10)Nb alloy plates after quenching from 600 °C: (**a**)—Ti-38Zr-8Nb, (**b**)—Ti-38Zr-9Nb, (**c**)—Ti-38Zr-10Nb.

**Figure 2:**
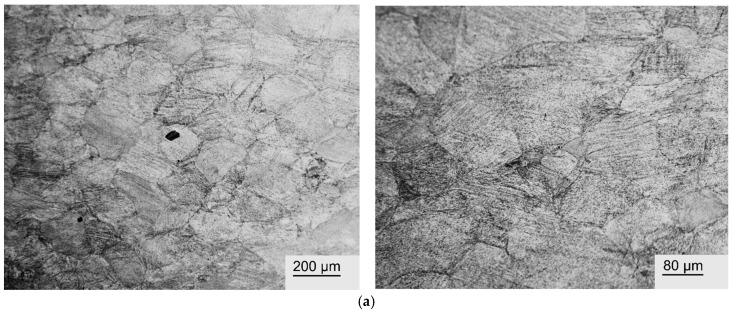

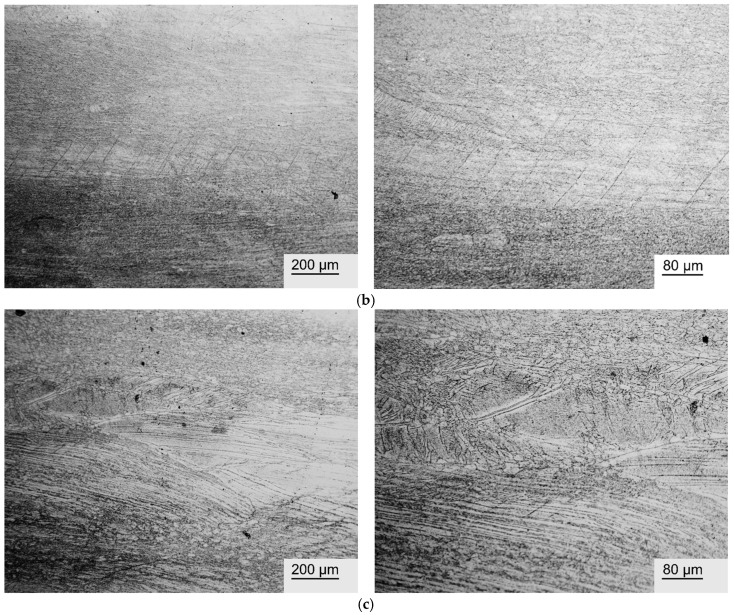
Microstructure of Ti-38Zr-(8-10) alloy plates Nb after quenching at 600 °C and annealing at 400 °C for 1 h in a vacuum: (**a**)–Ti-38Zr-8Nb, (**b**)—Ti-38Zr-9Nb, (**c**)—Ti-38Zr-10Nb.

Figure 1 and Figure 2 show photographs of the microstructure of Ti-38Zr-(8-10)Nb alloy plates after quenching at 600 °C and after quenching at 600 °C followed by annealing for 1 h at 400 °C.

The microstructure photographs show that hardening of the Ti-38Zr-8Nb alloy leads to recrystallization of the alloy and grain growth to sizes of 80–100 μm. Heating Ti-38Zr-(9-10)Nb alloys for hardening leads to the start of the recrystallization process with grain sizes up to 20 μm. During subsequent annealing at 400 °C, no noticeable recrystallization of the alloy occurred.

### 3.3. X-Ray Phase Analysis

**Figure 3:**
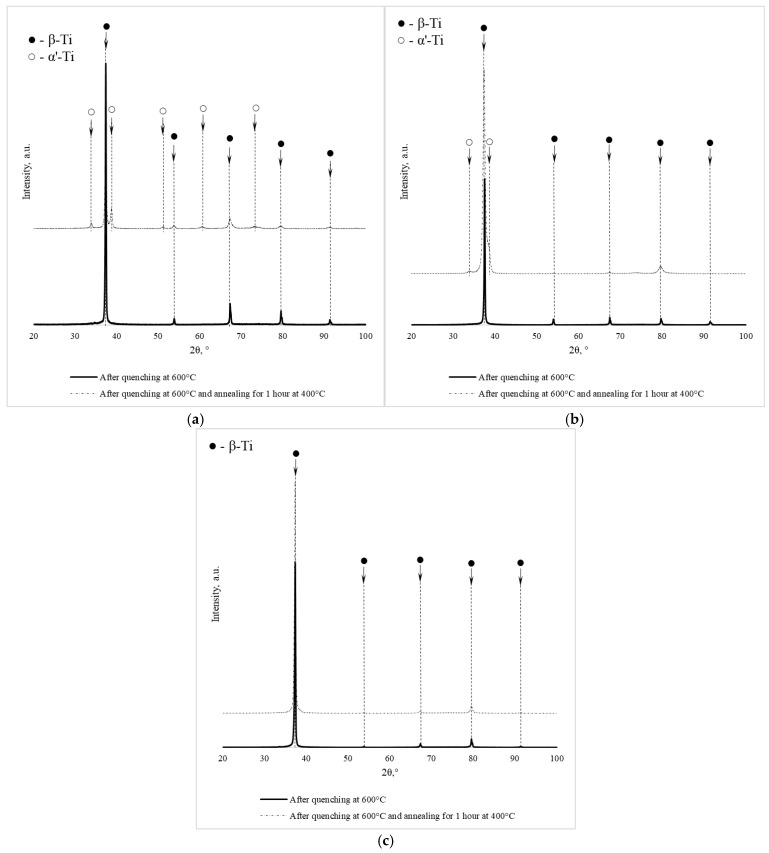
Diffractograms of Ti-38Zr-(8-10)Nb alloys: (**a**)—Ti-38Zr-8Nb, (**b**)—Ti-38Zr-9Nb, (**c**)—Ti-38Zr-10Nb.

The phase composition determined by X-ray diffraction for the plates subjected to rolling with quenching and rolling with quenching plus annealing is given in Table 3, while Figure 3 displays the diffractograms.

X-ray diffraction analysis (Figure 3, Table 3) showed that after quenching from 600 °C, all investigated Ti–38Zr–(8–10)Nb alloys exhibit a single-phase structure based on β-Ti with a BCC lattice. The absence of martensitic phases (α′, α″) and the ω-phase indicates that the addition of 8–10% Nb in combination with 38% Zr suppresses diffusionless transformations during quenching. According to the ternary Ti–Zr–Nb phase diagram, these compositions lie within the single-phase β region at the quenching temperature, with increasing Nb content shifting the alloy deeper into the β-phase stability field. This explains the complete retention of the β-solid solution upon cooling. Zirconium, acting as an isomorphous β-stabilizer, further extends the β-phase region [26].

Subsequent annealing at 400 °C for 1 h leads to partial decomposition of the metastable β-phase in the alloys with 8 and 9% Nb. This results in the formation of a two-phase structure consisting of a β matrix and precipitates of the α′-phase with a hexagonal lattice. The amount of α′-phase formed logically decreases with increasing niobium concentration: from 20% in the Ti–38Zr–8Nb alloy to 12% in Ti–38Zr–9Nb. This is in good agreement with the position of the compositions on the phase diagram: an increase in the β-stabilizer content lowers the polymorphic transformation temperature and reduces the equilibrium fraction of the α-phase at 400 °C.

In the Ti–38Zr–10Nb alloy, after similar annealing, the α′-phase was not detected—the phase composition remains purely β. This indicates that at 10% Nb, even annealing at 400 °C does not cause noticeable decomposition of the solid solution within the sensitivity limit of the XRD method. It is possible that at this β-stabilizer content, diffusion processes are slowed down, or the equilibrium amount of the α-phase at 400 °C is too small to be detected.

It is worth noting a slight increase in the lattice parameter of the β-phase after annealing in the alloys with 8 and 9% Nb (from 3.40 to 3.41 Å). This may be related to a change in the chemical composition of the β-solid solution due to the precipitation of the α′-phase: the α′-phase, enriched in titanium and zirconium, depletes the β-phase of these elements, increasing its relative niobium content, which leads to a small change in the lattice period. For the alloy with 10% Nb, where no decomposition occurs, the lattice parameter remains unchanged.

**Figure 4:**
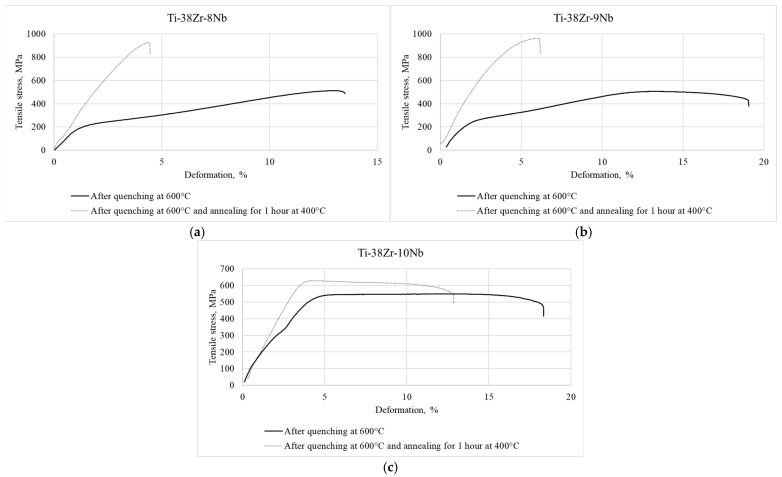
Tensile diagram of samples made of Ti-38Zr-(8-10)Nb alloys: (**a**)—Ti-38Zr-8Nb, (**b**)—Ti-38Zr-9Nb, (**c**)—Ti-38Zr-10Nb.

The diffraction patterns of the annealed alloys also show weak reflections (2–3 times above the background level), which may correspond to a finely dispersed ω-phase. The formation of the ω-phase during annealing of metastable β-titanium alloys is typical and often leads to embrittlement. The observed sharp drop in ductility (Figure 4a,b) correlates well with the possible precipitation of ω-particles [27], even if their amount is too small for reliable identification by XRD.

### 3.4. Mechanical Properties

Figure 4 displays typical tensile stress–strain diagrams obtained from Ti-38Zr-(8-10)Nb alloy specimens. The samples were examined in two conditions: after quenching from 600 °C, and after quenching from 600 °C with subsequent annealing at 400 °C for 1 h in vacuum.

The mechanical properties are presented in Table 4.

In the tensile stress–strain diagrams of the Ti-38Zr-(8-9)Nb alloys, yield plateaus are observed, which may indicate the occurrence of a phase transformation during loading and, consequently, the manifestation of the superelasticity effect in these alloys.

**Figure 5:**
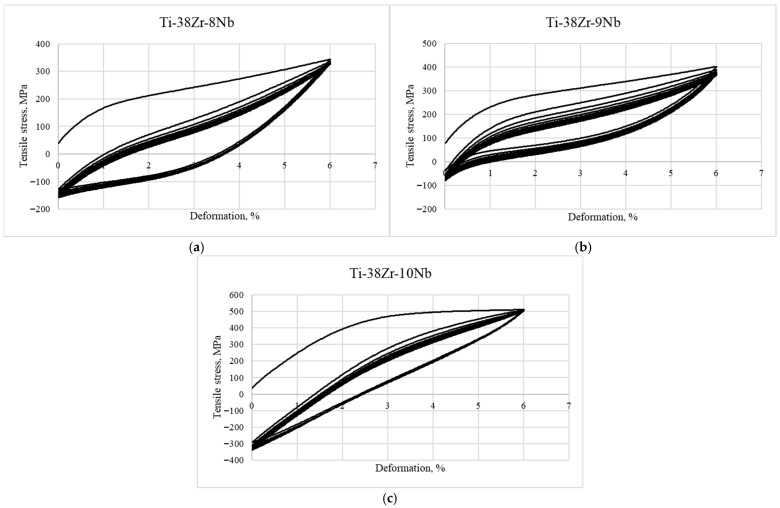
Cyclic tensile diagrams of Ti-38Zr-(8-10)Nb alloy after quenching from 600 °C: (**a**)—Ti-38Zr-8Nb, (**b**)—Ti-38Zr-9Nb, (**c**)—Ti-38Zr-10Nb.

As can be seen from Figure 5a,b, annealing leads to a sharp decrease in the ductility of the Ti-38Zr-(8-9)Nb alloys, accompanied by an increase in strength. The signs of superelastic behavior (the yield plateau) disappear. This may be associated with the precipitation of hard α (α′) and ω phases, as well as the stable β-phase, from the soft metastable β-metastable phase retained by quenching. In the alloy with 10% Nb, a significantly smaller amount of precipitates, likely without the ω-phase, forms, since the ductility level of this alloy remains at an acceptable level [27].

### 3.5. Superelastic Properties

To evaluate the superelastic properties, the alloys were cyclically loaded to 6% strain for 10 cycles. The strain–stress diagrams for the Ti-38Zr-(8-10)Nb alloys are shown in Figure 5. The negative stress values correspond to the compression region and bending of the specimen during the unloading stage of the induced plastic deformation.

Low-cycle fatigue tests with a strain amplitude of 6% showed that the best superelasticity (residual strain of ~1%) is achieved in the Ti-38Zr-9Nb alloy. This is attributed to the optimal stability of the metastable β-phase after quenching, which is determined by the Nb content.

In the Ti-38Zr-8Nb alloy, the β-phase is the least stable. Under 6% strain, the martensitic transformation (β→α″) is induced; however, due to its low stability, a large proportion of irreversible plastic deformation occurs, resulting in higher residual strain. The coarse grain size (80–100 μm) also contributes to strain localization.

In the Ti-38Zr-10Nb alloy, the β-phase is the most stable. At 6% strain, the martensitic transformation takes place to a very small extent. Deformation is accommodated predominantly elastically and through plastic flow of the β-phase; therefore, the superelasticity effect is less pronounced.

The Ti-38Zr-9Nb alloy occupies an optimal intermediate position. The stability of the β-phase is sufficient to suppress irreversible martensitic transformation during quenching, yet under load, the reversible martensitic transformation proceeds effectively, providing shape recovery. The fine grain size (approximately 20 μm) promotes uniform deformation.

### 3.6. Young’s Modulus

Nanoindentation was used to measure the Young’s modulus of the Ti-38Zr-(8-10)Nb alloys in two conditions: after quenching from 600 °C and after subsequent annealing at 400 °C for 1 h. The measured values are listed in Table 5.

The greatest change in elastic modulus after annealing is observed in the Ti-38Zr-8Nb alloy: from 78 ± 5 to 96 ± 4 GPa. Young’s modulus in the Ti-38Zr-10Nb alloy after annealing remains virtually unchanged at around 80 GPa.

### 3.7. Fractographic Studies

**Figure 6:**
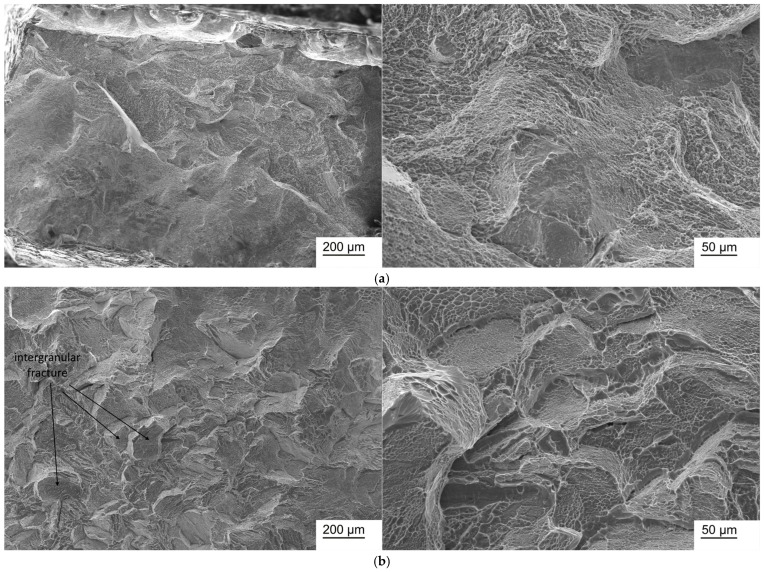
Micrographs of a fracture of a Ti-38Zr-8Nb alloy sample: (**a**)—after quenching from 600 °C; (**b**)—after quenching from 600 °C and annealing at 400 °C for 1 h in a vacuum.

**Figure 7:**
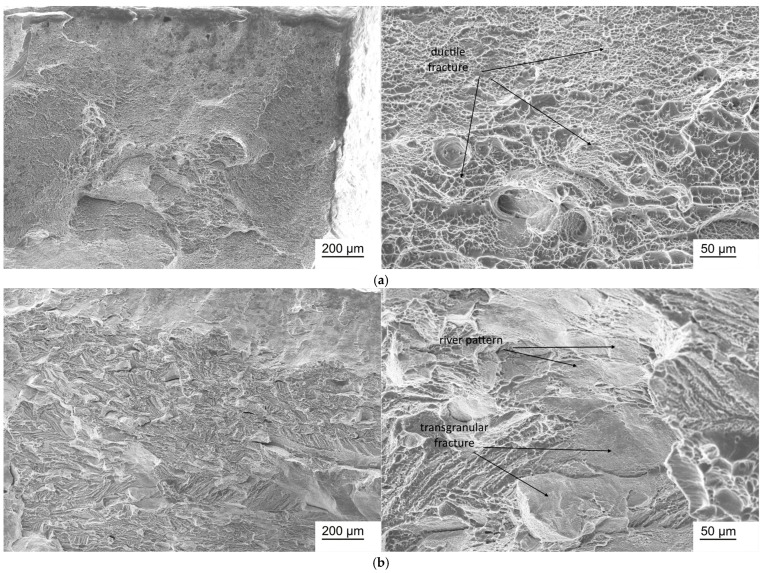
Micrographs of a fracture of a Ti-38Zr-9Nb alloy sample: (**a**)—after quenching from 600 °C; (**b**)—after quenching from 600 °C and annealing at 400 °C for 1 h in a vacuum.

**Figure 8:**
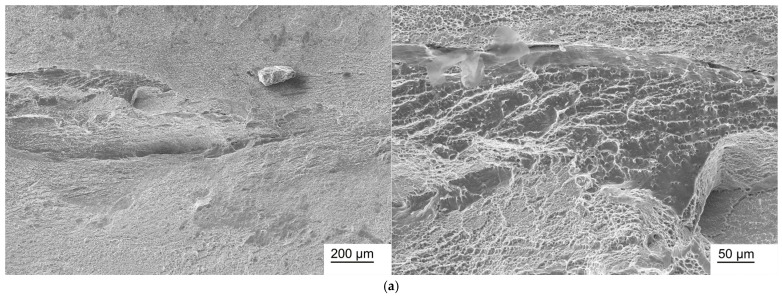

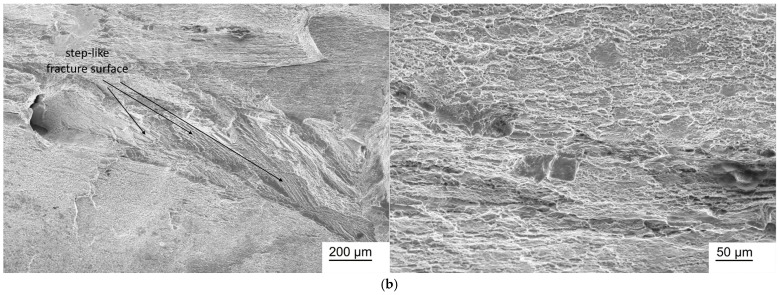
Micrographs of a fracture of a Ti-38Zr-10Nb alloy sample: (**a**)—after quenching from 600 °C; (**b**)—after quenching from 600 °C and annealing at 400 °C for 1 h in a vacuum.

Fractographic studies of Ti-38Zr-(8-10)Nb alloy samples in the state after quenching from 600 °C and after annealing at 400 °C for 1 h in a vacuum are presented in Figure 6, Figure 7 and Figure 8.

In the Ti-38Zr-(8-10)Nb alloys after quenching, a transgranular ductile fracture is observed, characterized by numerous rounded/elliptical depressions—dimples—formed by the nucleation of microvoids around inclusions/phases, the growth and coalescence of which during deformation leads to the formation of such dimples. In the Ti-38Zr-8Nb alloy after annealing, the fracture surface contains regions with a “river pattern” characteristic of brittle fracture, as well as regions with a dimpled relief. Moreover, the presence of flat facets on the fracture surface suggests that failure occurred partially along grain boundaries (intergranular fracture, with grain boundaries indicated by arrows in Figure 7b), which correlates with the microstructural observations showing that the Ti-38Zr-8Nb alloy has a recrystallized coarse-grained structure. In the Ti-38Zr-9Nb alloy after annealing, both dimpled areas of ductile transgranular fracture and cleavage facets with a “river pattern” (Figure 7b) are present; the intergranular character of the fracture is significantly less pronounced. In the Ti-38Zr-10Nb alloy after annealing, a predominantly dimpled transgranular fracture relief is observed, although areas with stepped cleavage are also present (Figure 8b).

## 4. Conclusions

1. In Ti-38Zr-(8-10)Nb alloys, quenching from 600 °C retains the β-phase of Ti. In alloys with (8-9)Nb, this is a metastable β-phase, as evidenced by its superelastic behavior under cyclic tensile loading. Annealing at 400 °C leads to a clear decomposition of the retained high-temperature β-phase in Ti-38Zr-(8-9)Nb alloys into β- and α’-phases. Furthermore, based on mechanical tests, it can be assumed that the precipitation of the brittle ω-phase occurs simultaneously with the precipitation of the α’-phase.
2. Annealing at 400 °C leads to severe embrittlement of the Ti-38Zr-(8-9)Nb alloys (ductility drops from ~15% to 0.7–2.5%, respectively) with a rise in strength (from 500 MPa to 1010 MPa). For the Ti-38Zr-10Nb alloy, ductility also decreases, but to acceptable levels (from ~14% to ~10%), accompanied by an increase in strength from 520 to 630 MPa.
3. The Young’s modulus of the Ti-38Zr-(8-10)Nb alloys after quenching is ~80 GPa. After annealing, it increases to 95 GPa for alloys with (8-9)Nb, while for the 10Nb alloy it remains at ~80 GPa.
4. Among the investigated Ti-38Zr-(8-10)Nb alloys, the optimal combination of properties after quenching is achieved with a niobium content of 9 at.%. This is attributed to the optimal stability of the β-phase and the formation of the most favorable microstructure for the manifestation of superelasticity.

## Figures and Tables

**Table 1:** The compositions of the alloys.

Alloy	Ti, at. %	Zr, at. %	Nb, at. %
Ti-38Zr-8Nb	54.2 ± 0.1	37.4 ± 0.1	8.3 ± 0.1
Ti-38Zr-9Nb	53.6 ± 0.1	37.2 ± 0.1	9.2 ± 0.1
Ti-38Zr-10Nb	52.7 ± 0.1	37.2 ± 0.1	10.0 ± 0.1

**Table 2:** Quantitative characteristics of impurities.

Alloy	O, Mass %	N, Mass %	H, Mass %	C, Mass %	S, Mass %
Ti-38Zr-8Nb	0.070 ± 0.005	0.0096 ± 0.004	0.0067± 0.001	0.025 ± 0.005	0.0039 ± 0.0009
Ti-38Zr-9Nb	0.016 ± 0.005	0.0029 ± 0.004	0.0081 ± 0.001	0.017 ± 0.005	0.0048 ± 0.0009
Ti-38Zr-10Nb	0.03 ± 0.005	0.0029 ± 0.004	0.0068 ± 0.001	0.014 ± 0.005	0.0055 ± 0.0009

**Table 3:** Phase composition of Ti-38Zr-(8-10)Nb alloy plates.

Alloy	State	Phase Composition	Crystal Lattice Parameters, Å
Ti-38Zr-8Nb	After quenching from 600 °C	β-Ti, 100%	a = 3.40 ± 0.01
	After quenching from 600 °C and annealing for 1 h at 400 °C	β-Ti, 80%	a = 3.41 ± 0.01
		α′-Ti, 20%	a = 3.07 ± 0.01 b = 4.84 ± 0.01
Ti-38Zr-9Nb	After quenching from 600 °C	β-Ti, 100%	a = 3.40 ± 0.01
	After quenching from 600 °C and annealing for 1 h at 400 °C	β-Ti, 88%	a = 3.41 ± 0.01
		α′-Ti, 12%	a = 3.09 ± 0.01 b = 4.83 ± 0.01
Ti-38Zr-10Nb	After quenching from 600 °C	β-Ti, 100%	a = 3.40 ± 0.01
	After quenching from 600 °C and annealing for 1 h at 400 °C	β-Ti, 100%	a = 3.40 ± 0.01

**Table 4:** Mechanical properties.

Alloy	State	Relative Elongation δ, %	Tensile Strength σ_u_, MPa
Ti-38Zr-8Nb	After quenching from 600 °C	15.2 ± 1.3	505 ± 21
	After quenching from 600 °C and annealing for 1 h at 400 °C	0.7 + 0.2	1010 + 9
Ti-38Zr-9Nb	After quenching from 600 °C	15.4 ± 0.2	502 ± 6
	After quenching from 600 °C and annealing for 1 h at 400 °C	2.5 ± 0.1	1009 ± 43
Ti-38Zr-10Nb	After quenching from 600 °C	14.2 ± 0.9	523 ± 36
	After quenching from 600 °C and annealing for 1 h at 400 °C	9.7 ± 1.1	629 ± 31

**Table 5:** Young’s modulus.

Alloy	After Quenching from 600 °C, GPa	After Quenching from 600 °C and Annealing for 1 h at 400 °C, GPa
Ti-38Zr-8Nb	78 ± 5	96 ± 4
Ti-38Zr-9Nb	83 ± 1	95 ± 5
Ti-38Zr-10Nb	80 ± 2	79 ± 1

## Data Availability

The original contributions presented in this study are included in the article. Further inquiries can be directed to the corresponding author.
